# Transverse electron momentum distribution in tunneling and over the barrier ionization by laser pulses with varying ellipticity

**DOI:** 10.1038/srep19002

**Published:** 2016-01-07

**Authors:** I. A. Ivanov, A. S. Kheifets, J. E. Calvert, S. Goodall, X. Wang, Han Xu, A. J. Palmer, D. Kielpinski, I. V. Litvinyuk, R. T. Sang

**Affiliations:** 1 Center for Relativistic Laser Science, Institute for Basic Science, Gwangju 500-712, Republic of Korea; 2 Research School of Physics and Engineering, The Australian National University, Canberra ACT 0200, Australia; 3 School of Natural Sciences and Centre for Quantum Dynamics, Griffith University, Nathan QLD 4111, Australia

## Abstract

We study transverse electron momentum distribution in strong field atomic ionization driven by laser pulses with varying ellipticity. We show, both experimentally and theoretically, that the transverse electron momentum distribution in the tunneling and over the barrier ionization regimes evolves in a qualitatively different way when the ellipticity parameter describing polarization state of the driving laser pulse increases.

A highly non-linear interaction of ultra-short light pulses with matter enabled studying electron dynamics on the atomic time scale and facilitated emergence of the attosecond science^1^. In addition, strong field atomic ionization proved itself a potent tool to interrogate atomic and molecular orbital structure via high order harmonic radiation^2^, tunneling and diffraction^3^ or tunneling and momentum imaging^4^. This utility of strong field atomic ionization is based on the electric field of the laser pulse bending the Coulomb barrier and letting a bound electron to tunnel out from an atom or a molecule.

Within the premise of the Keldysh theory^5^, this tunneling regime of strong field ionization corresponds to a small value of the adiabaticity parameter 
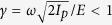
 defined via the frequency *ω* and the strength *E* of the laser field and the ionization potential *I*_*p*_ of the target atom (the atomic units are used in the paper unless otherwise specified). A finer distinction arises when one realizes that the Keldysh theory in its original form is not applicable for very high field strengths exceeding the over the barrier (OBI) limit. The OBI regime was first observed in^6^ (see also^7^ for a detailed review). The Keldysh theory in its original form fails in the OBI regime because there is a classical escape trajectory for an electron. One cannot, therefore, rely on the saddle point method that Keldysh employed in his original work. The so-called Keldysh-Faisal-Reiss (KFR) theory^8^,^9^ must be used instead to describe the OBI regime. We also note that the Keldysh theory^5^ and its subsequent developments and generalizations^10^,^11^,^12^,^13^ describe the quasistatic limit of small laser pulse frequencies. In strict terms, the Keldysh approach provides a leading-order term in the asymptotic expansion of the ionization rate, a systematic way to obtain higher order terms is described in^14^,^15^.

Despite the fact that underlying physics is very different in the two regimes (a classically forbidden trajectory for tunneling and a classically allowed trajectory for OBI), the energy spectra and electron angular distributions as given by these two theories are not dissimilar. In this work, we demonstrate that the transverse electron momentum distribution (TEMD) is a measurable quantity that is qualitatively different in the tunneling and the OBI regimes. This distribution (also known in the literature as the lateral electron momentum distribution^16^) gives the probability to detect a photoelectron with a given value of the momentum component *p*_⊥_ perpendicular to the polarization plane of the laser radiation. We are concerned with the TEMD observed in the experiment which is measured at the detector after the laser pulse has finished. In the tunneling regime, TEMD exhibits a cusp-like structure due to the Coulomb focusing effect at *p*_⊥_ = 0 for linear polarization^17^, and a Gaussian-like structure predicted by the Keldysh theory for circular polarization^4^. We studied this transition from the cusp-like to the Gaussian structures in detail in the tunneling regime^18^, and interpreted this transition as a gradual diminishing of the role of the Coulomb effects with growing ellipticity of the laser pulse. Further study of the role of the Coulomb focusing effects was reported in^19^. We shall see below that the situation is quite different in the OBI regime, where the TEMD always has a cusp regardless of the value of the ellipticity parameter. As a result of this qualitatively different behavior of the TEMD, one can clearly distinguish the tunneling and OBI regimes. This is an important result since the TEMD conveys information about the fine details of the strong field ionization process^20^,^21^. One such detail is the electron velocity distributions at the moment of time when ionization occurs, which is often used in various models of strong field ionization. The omnipresence of the cusp in the OBI regime also makes it unsuitable for momentum imaging proposed in^4^,^16^.

## Methods

As the case study, in the present work we select two markedly different atomic species: the argon atom in the ^1^*S*_0_ ground state and the neon atom in the ^3^*P*_2_ metastable state with the ionization potentials of 15.76 eV and 5.07 eV, respectively. An estimate for the critical field corresponding to the onset of OBI can be found from the equation 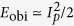 which follows from considering the hydrogen atom placed in an external field using the parabolic coordinates^22^. This rough estimate, which does not account for the above-barrier reflection^7^ and the Stark shift, places the OBI onset of at Ar 9.9 × 10^14^ W/cm^2^ while for Ne^*^ this onset starts at 1.1 × 10^13^ W/cm^2^. This comfortable two orders of magnitude difference allows to drive these targets to the tunneling and OBI regimes with comparable laser intensities in the same experimental set up (Ar@ 4.8 × 10^14^ W/cm^2^ and metastable Ne^*^ @ 2 × 10^14^ W/cm^2^, both corresponding to a similar adiabaticity parameter 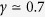 at 800 nm). To our knowledge the only known momentum imaging experiment in similar OBI regime was reported on Li^23^.

### Experimental setup and procedure

**Figure 1:**
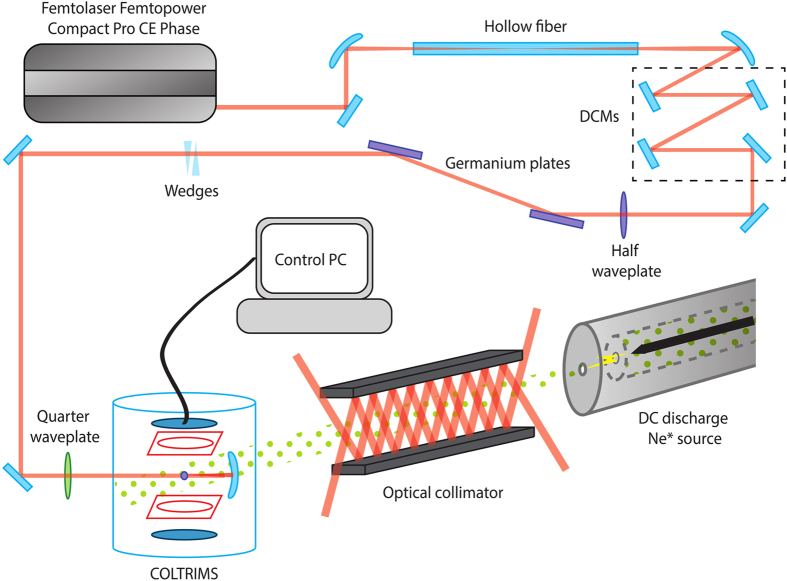
Schematic representation of the experiment.

A schematic representation of the experiment is shown in Fig. 1. The ultrafast light pulses are produced by a commercially available chirped-pulse amplification laser system (Femtolasers, Femtopower Compact Pro CE Phase). The light pulses are generated, stretched, amplified and then compressed in the system. The germanium plates are positioned at the Brewster’s angle and used together with the half waveplate to provide power control of the beam with a linear polarisation. The glass wedges are used to provide final adjustment to the group dispersion delay of the laser pulse, and hence control the pulse length. The quarter waveplate is used to provide a degree of ellipticity to the laser beam. The pulse repetition rate is 1 kHz. Typically, the system is run a pulse duration of 6.3 fs, as measured with an autocorrelator. The laser can produce maximum pulse energies of approximately 450 *μ*J. The pulse train is focused down to a spot size of ≈7.25 *μ*m radius (FWHM) at the interaction region of the electron detection device. The electron detection device is a reaction microscope (REMI). In the interaction region of the REMI, the laser pulse ionizes atoms from a target atom beam. Photoionised electrons are directed towards one detector by utilising an electric field provided by an array of copper plates. Likewise, positive ions from interaction events are similarly directed towards a separate detector, however this data is not used for this experiment. The electron detector is a position dependent delay-line time of the flight detector. This allows for the determination of the momentum vectors of the ionized electrons. More information on the experimental setup can be found in^24^. The Ar beam is provided by a cold gas jet source. Metastable ^3^*P*_2_ neon atoms are produced by a gas discharge source, which uses a DC discharge across a supersonic gas expansion region to excite approximately 1% of neon atoms in a gas jet into the correct state. The flux of metastable neon atoms is improved by the optical collimation technique that take advantage of the 640 nm closed optical transition to the ^3^*D*_3_ state. Further details of this gas source can be found in^25^,^26^.

The experimental results are taken for metastable ^3^*P*_2_ neon as follows. Firstly, the pressure in the REMI chamber is reduced below 5 × 10^−10^ Torr, the limit of the vacuum gauges. At this point, neon gas is fed into the chamber through the cold jet gas source. Ion momentum distributions of ground state neon ionisation are taken at varying laser powers, for both linear and circular polarisations. This data is used to create a calibration curve for the intensity of the laser pulse at the interaction region as per the method described in^27^. Next, the metastable neon source is engaged and the intensity of the laser pulse is set to 2 × 10^14^ W/cm^2^. We have determined that 90 percent of the measured ionization rate comes from OB ionization which has been confirmed though the measurement of the momentum distributions of ionized electrons in the plane of the laser polarization using circularly polarized light. The ellipticity parameter of the laser pulses is set to *ε* = 0 with the quarter waveplate, by aligning the fast axis of the waveplate with the polarisation axis off the germanium plates. The REMI is set to integrate results over 1.8 × 10^6^ laser pulses. The transverse electron momentum data is extracted from the software controller of the REMI as a number density map. This information is binned, integrated and normalised to give a plot of ionisation rate (*W*(*p*_⊥_)) as a function of transverse electron momentum (*p*_⊥_). This process is repeated for *ε* = 0.42 and *ε* = 1.

Experimental results for Ar are taken in a similar way, replacing the metastable ^3^*P*_2_ neon atoms from the DC discharge source with argon atoms from the cold gas jet source.

### Theoretical methods

Our theoretical results are obtained by solving the time-dependent Schrödinger equation (TDSE):


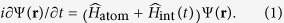


To describe the field-free Ar and metastable Ne^*^ atoms, we used effective one-electron potentials^28^. The interaction of the atom with the laser pulse is described in the velocity form of the interaction operator:


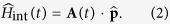


where **A**(*t*) is the vector potential of the laser pulse. The laser pulse is elliptically polarized and propagates along the *z*-direction which is assumed to be the quantization axis:


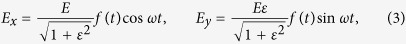


where *ε* is the ellipticity parameter. The function *f*(*t*) = sin^2^(*πt*/*T*_1_), with *T*_1_ being the total pulse duration, is used to represent the pulse envelope. For the Ar atom, the field strength was *E* = 0.1171 a.u. corresponding to the experimental peak intensity of 4.8 × 10^14^ W/cm^2^. For the metastable Ne^*^ atom, *E* = 0.0756 a.u. with the peak intensity of 2 × 10^14^ W/cm^2^. The carrier wavelength *λ* = 800 nm and the FWHM of 6 fs were the same for Ar and Ne^*^.

To solve the TDSE we employ the strategy used in the previous works^18^,^29^,^30^. The solution of the TDSE is represented as a partial waves series:


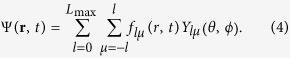


The radial part of the TDSE is discretized on the grid with the stepsize *δr* = 0.1 a.u. in a box of the size *R*_max_ = 400 a.u. The maximum orbital momentum in Eq. (4) was restricted to *L*_max_ = 60. Convergence with respect to variation of *δr*, *R*_max_ and *L*_max_ was carefully monitored. The matrix iteration method^31^ was used to propagate TDSE in time. Ionization amplitudes *a*(**p**) were obtained by projecting the solution of the TDSE after the end of the pulse on the set of the ingoing scattering states 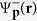 of the target atom. The TEMD *W*(*p*_⊥_) was calculated as


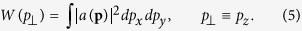


## Results

**Figure 2:**
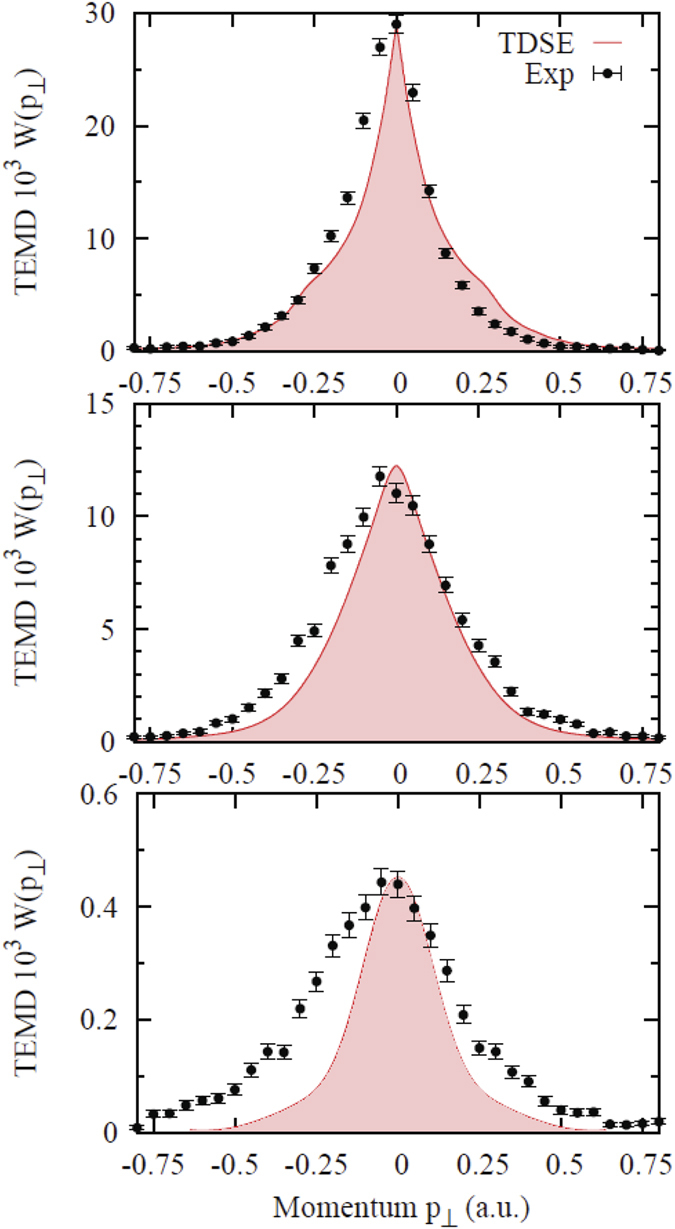
TEMD of Ar (multiplied by 10^3^) for ellipticity parameters *ε* = 0, 0.42, and 1 (from top to bottom). TDSE calculation is shown by a (red) solid curve (shaded for a clearer appearance), experimental data are plotted with error bars. The peak intensity of the laser pulses is 4.8 × 10^14^ W/cm^2^.

Experimental and theoretical TEMD results for Ar are shown in Fig. 2. A general trend of the calculated TEMD with increase of ellipticity is very similar to that reported previously for the simulations of the hydrogen atom^18^. The cusp-like structure is present for linear polarization and it gradually evolves into a Gaussian distribution as the ellipticity parameter increases. Agreement between the theory and experiment is good for linear polarization but gradually deteriorate with an increase of ellipticity.

**Figure 3:**
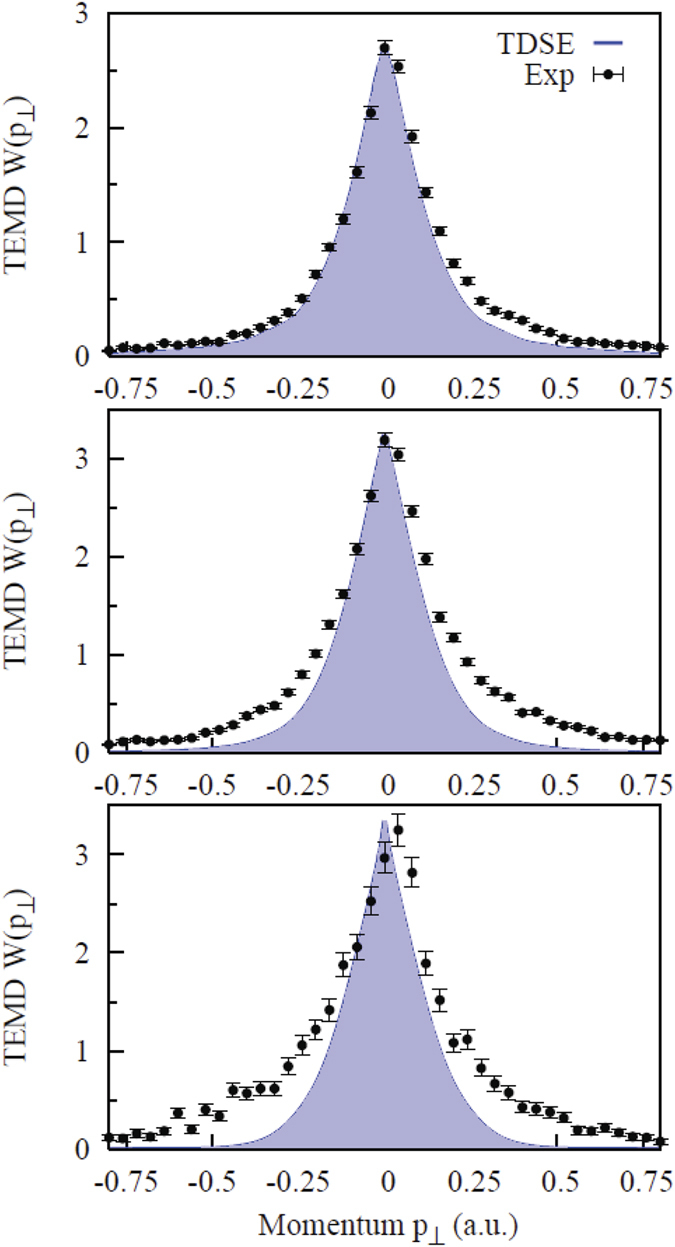
TEMD of metastable Ne^*^ for ellipticity parameters *ε* = 0, 0.42, and 1 (top to bottom). TDSE calculation is shown by a (blue) solid curve (shaded for a clearer appearance), experimental data are plotted with error bars. The peak intensity of the laser pulses is 2 × 10^14^ W/cm^2^.

Figure 3 presents the theoretical and experimental TEMD results for metastable Ne^*^. In this target atom, the TEMD evolution with the ellipticity parameter is greatly reduced with the cusp clearly present even for the circularly polarized pulse. Similar to Ar, agreement between the theory and experiment progressively worsens from the top to bottom panels.

To analyze the cusp in more detail, we zoomed in on the narrow range of momenta 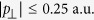 and analyzed the function *V*(*p*_⊥_) = ln *W*(*p*_⊥_) in this interval. For the TEMD *W*(*p*_⊥_) to have a cusp, *V*(*p*_⊥_) should have an infinite derivative of some order and have an expansion near *p*_⊥_ = 0:


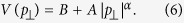


Such expansions, in fact, reproduce 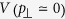 very well as was shown in^18^.

**Figure 4:**
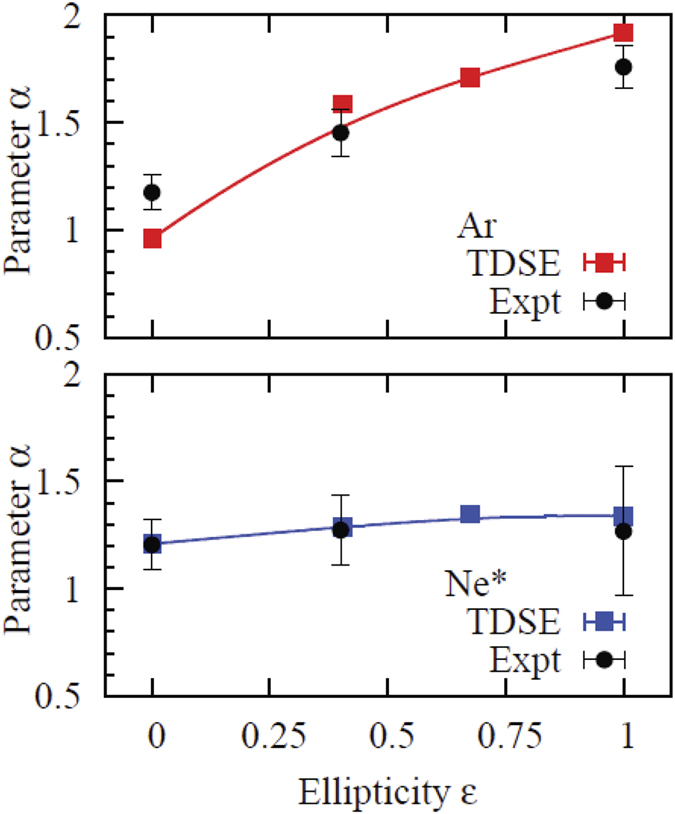
The fitting parameter *α* in Eq. (6) as a function of the ellipticity parameter *ε* for Ar (top) and Ne^*^ (bottom). TDSE results are shown with squares (a smooth solid line is to guide the eye). The experimental data points are shown with error bars.

The same functional form (6) was used to fit both the theoretical and experimental data for the ground state Ar and the metastable Ne^*^ in the whole range of ellipticities by considering the coefficients *A*, *B*, *α* as fitting parameters. The most essential *α* parameters are shown in Fig. 4 for Ar (top) and Ne^*^ (bottom). Both theoretical and experimental values are shown with error bars which represent the fitting error. For the theoretical data, this error does not exceed a fraction of a percent and is not visible on the scale of the figure.

## Discussion

The *α* parameters shown on the top and bottom panels of Fig. 4 demonstrate a qualitatively different behavior as functions of the ellipticity. For the Ar atom, the *α* parameter grows with *ε* reaching the value close to 2 for circular polarization. This implies that TEMD becomes close to a Gaussian 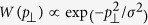 with the Gaussian width related to the fitting parameter 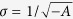. The corresponding numerical values of 0.25 ± 0.002 and 0.28 ± 0.02 for the TDSE and experiment, respectively, are close to the experimental values reported in^4^ for comparable field intensities. In the meantime, the *α* parameters for the metastable Ne^*^ atom remain essentially flat, indicating that a cusp-like behavior is present for all *ε* in the range from linear to circular polarization. In this case, extraction of the Gaussian width parameter is not possible even for the circular polarization. Several TDSE calculations performed for different field intensities did not show any considerable variation of the cusp width. However, the Gaussian width varies with the field strength as 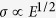. This may explain, at least partially, deviation between the measured and calculated TEMD with circular polarization due to the variation of the field strength across the laser-atom interaction region while the calculation was performed at a single nominal field intensity. TDSE calculations for the polarization of the driving pulse other than linear are genuinely 3D-calculations which makes them quite time-consuming, thereby effectively precluding focal volume and carrier-envelope phase averaging of theoretical data.

While the Ar case shows the behavior qualitatively similar to that found previously for hydrogen^18^, the metastable Ne^*^ presents a different trend, with the cusp never disappearing completely. In this case, a simplified description based on the Keldysh theory is never correct even qualitatively. This qualitative difference can be explained by the different ionization regimes for Ar and metastable Ne^*^.

**Figure 5:**
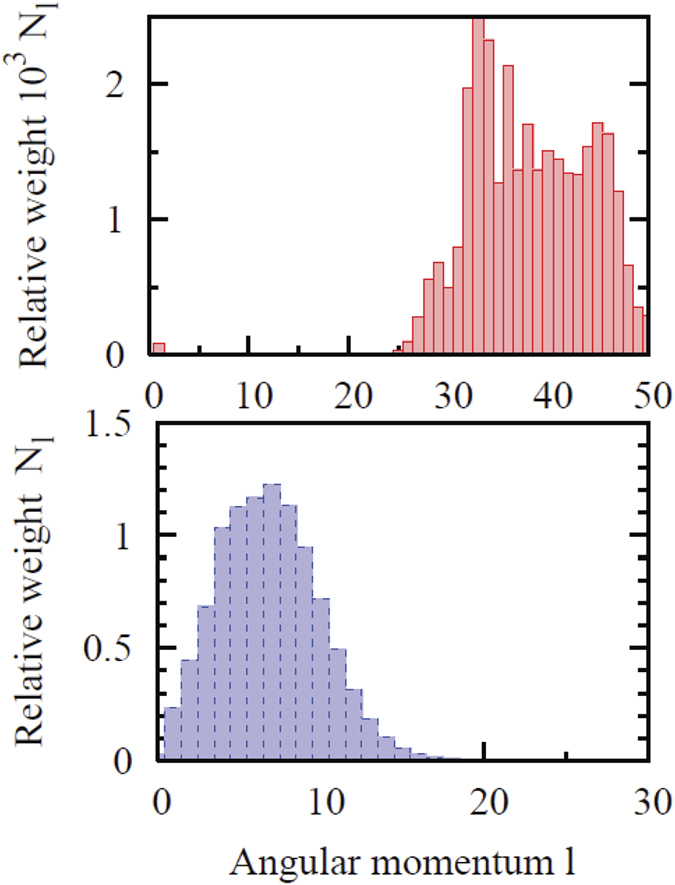
Angular momentum distribution *N*_*l*_ for Ar@ 4.8 × 10^14^ W/cm^2^ (top) and metastable Ne^*^ @ 2 × 10^14^ W/cm^2^, bottom). Laser field is circularly polarized.

The TEMD cusp disappearance with increasing *ε* can be related to a dramatic change of the angular momentum composition of the ionized electron wave function^18^. This composition is characterized by the distribution of the norm *N*_*l*_ of the wave function obtained if only the terms with spherical harmonics of rank *l* are retained in expansion (4). For a tunneling process this distribution is shifted towards larger *l* with increasing pulse ellipticity parameter. Indeed, tunneling can be viewed as a non-resonant absorption of a large number of photons. Absorption of a photon from the circularly polarized wave increases the magnetic quantum number by one unit. This leads to a prevalence of high angular momenta in the partial wave expansion (4). High angular momenta create large centrifugal barrier preventing recolliding electron trajectories, thereby suppressing the Coulomb focusing effects. The cusp, therefore, vanishes for polarization close to circular, as in the case of Ar reported here, or hydrogen^18^. The situation with the metastable Ne^*^ is completely different. OBI dominates in this case, and since OBI is essentially a distortion of the atomic potential to the degree, that there is effectively a zero barrier to the continuum, the atom does not have to absorb many photons to become ionized. The distribution *N*_*l*_, therefore, is peaked at lower values of the angular momenta. That this is indeed the case can be seen in Fig. 5, where the distributions *N*_*l*_ are shown for Ar for the intensity of 4.8 × 10^14^ W/cm^2^ (tunneling) and Ne^*^ for the field intensity of 2 × 10^14^ W/cm^2^ (the OBI regime). Smaller angular momenta enhance the area near the origin where the Coulomb focusing effect is strongest. Larger angular momentum components are repelled from the origin due to the centrifugal barrier. Hence in the former case the cusp is always present whereas in the latter case it gradually vanishes. This corresponds to a classical trajectory starting from the origin whereas a tunneling trajectory starts at the point of exit from the tunnel. In the OBI regime the electron’s classical trajectory starts at the ion core regardless of the polarization of the laser pulse, which may be enough for the efficient Coulomb focusing even if the trajectory never returns to the core.

To summarize, we described an effect of bending the Coulomb barrier of the atom on the transverse electron momentum distribution (TEMD) in strong field ionization in the tunneling regime. This fundamental effect, which should be present in any atomic or molecular target, is measured experimentally and modeled theoretically in two markedly different species: the ground state Ar and metastable Ne^*^. The effect is substantial, it has never been described or observed before and it enables a clear distinction between the tunneling and OBI regimes in the experiment. Also, it has to be taken into account when using TEMD data to interrogate electronic orbitals of the target.

Finally, we note that the cusp disappearance in the case of circular polarization may seem to follow from a classical consideration. Indeed, in the circularly polarized field, the two orthogonal field components drive the photoelectron away from the ionized core thus reducing the Coulomb focusing effect. This classical consideration, however, fails to explain qualitatively different TEMD behavior in the tunneling and OBI regimes observed in the present study.

## Additional Information

**How to cite this article**: Ivanov, I. A. *et al*. Transverse electron momentum distribution in tunneling and over the barrier ionization by laser pulses with varying ellipticity. *Sci. Rep*. **6**, 19002; doi: 10.1038/srep19002 (2016).
